# *QuickStats*: Percentage* of Adults Aged ≥18 Years Who Have Difficulty Hearing Even When Using a Hearing Aid,^†^ by Age Group — National Health Interview Survey, United States, 2020^§^

**DOI:** 10.15585/mmwr.mm7112a5

**Published:** 2022-03-25

**Figure:**
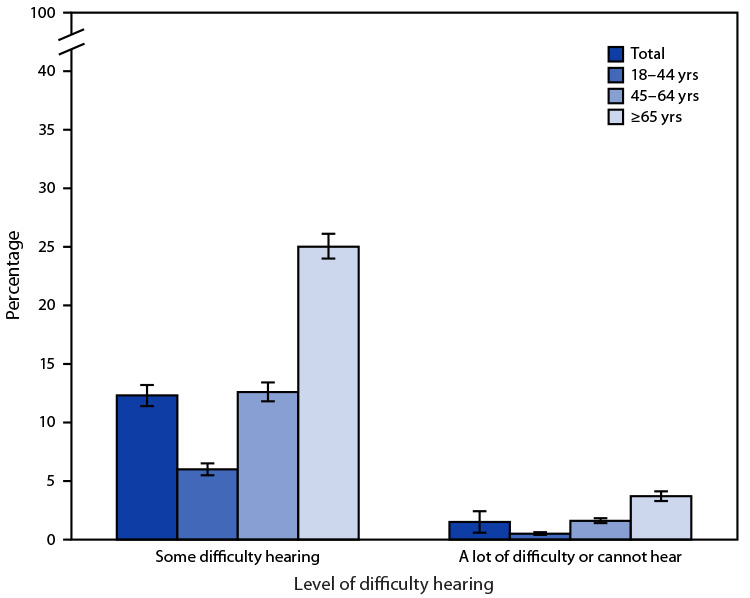
In 2020, 12.3% of adults aged ≥18 years had some difficulty hearing even when using a hearing aid and 1.5% had a lot of difficulty or could not hear at all. The percentage of adults who had some difficulty hearing increased with age, from 6.0% among those aged 18–44 years, to 12.6% among those aged 45–64 years, and to 25.0% among those aged ≥65 years. The percentage of adults who had a lot of difficulty hearing or were unable to hear at all also increased with age, from 0.5% among those aged 18–44 years, to 1.6% among those aged 45–64 years, and to 3.7% among those aged ≥65 years.

**Source:** National Center for Health Statistics, National Health Interview Survey, 2020. https://www.cdc.gov/nchs/nhis.htm

